# Downsizing a pullulanase to a small molecule with improved soluble expression and secretion efficiency in *Escherichia coli*

**DOI:** 10.1186/s12934-015-0403-5

**Published:** 2016-01-14

**Authors:** Ana Chen, Yang Sun, Wei Zhang, Feng Peng, Chunjun Zhan, Meng Liu, Yankun Yang, Zhonghu Bai

**Affiliations:** National Engineering Laboratory for Cereal Fermentation Technology, Jiangnan University, Wuxi, 214122 China; School of Biochemical Engineering, Anhui Polytechnic University, Wuhu, 241000 China; The Key Laboratory of Industrial Biotechnology and The Key Laboratory of Carbohydrate Chemistry and Biotechnology, Ministry of Education, School of Biotechnology, Jiangnan University, Wuxi, 214122 China

**Keywords:** Pullulanase, N-terminal domain truncation, Soluble expression level, Secretion efficiency, Cell lysis

## Abstract

**Background:**

Significant challenges, including low expression and extracellular secretion of soluble protein, are encountered in expressing and purifying *Bacillus acidopullulyticus* pullulanase (BaPul) in *Escherichia coli*.

**Methods:**

An N-terminal domain truncation was adopted to facilitate BaPul variant expression and/or secretion.

**Results:**

BaPul possesses a complex modular architecture that consists of CBM41-X45a-X25-X45b-CBM48-GH13. The activities of M1 (ΔCBM41) and M5 (ΔCBM41ΔX25) variants were 2.9- and 2.4-fold that of wild-type (WT) enzyme, respectively. The enhanced expression of soluble protein is the main reason for these improved activities. PelB-M1 and PelB-M5 were transported to the periplasmic space, where PelB is part of the PelB-pET28a(+) construct, and PelB-M3 (ΔX25) and PelB-WT variants were largely retained in the cytoplasm. After fermentation, about 56.6 and 93.4 % of the total activity of PelB-M1 and PelB-M5 were transferred to the periplasm, respectively, followed by cell lysis and leakage of the partial enzyme into the extracellular medium. The optimal temperature and pH for purified preparations of M1, M3, and M5 were similar to those of the WT enzyme. In a starch saccharification reaction, the dextrose equivalents of M1, M3, and M5 proteins were 94.7, 94.5, and 93.1 %, respectively, which were also essentially identical to that of WT (93.6 %).

**Conclusion:**

The deletion of CBM41 and/or X25 domain did not affect the enzyme application, and the truncated variants were more highly expressed and secreted in *E. coli*. Thus, the truncated variants may be more suitable for industrial applications.

## Background

Pullulanase is a debranching enzyme that can degrade the α-1,6-linkages of pullulan, amylopectin, and other branched polysaccharides [1]. In the grain industry, pullulanase is often used with glucoamylase or β-amylase to produce high glucose and maltose syrups [2, 3]. In addition to increasing the sugar yield, pullulanase reduces reaction time, allows for high substrate concentrations, and facilitates maltose production with 50 % of the normal glucoamylase concentration [4, 5]. The reverse synthesis action of pullulanase was verified at high substrate concentration. The reversibility of hydrolysis can be used to obtain polysaccharides with specific structures and functions [6, 7], which have potential applications in pharmaceutical industry. Pullulanases are widely distributed among animals, plants, fungi, and bacteria [8]. *Bacillus acidopullulyticus* pullulanase (BaPul) is the most practical easy-to-work debranching enzyme because its optimum temperature (60 °C) and pH (5.0) are suitable for the saccharification process used in industrial production [9].

The use of wild-type (WT) strains, such as *B. acidopullulyticus*, *B. deramificans*, and *B. naganoensis*, for the industrial production of pullulanases is limited because of their low productivity. Attempts have been made to overexpress pullulanase-encoding genes heterogeneously by using recombinant DNA technology. *Escherichia coli*, *Bacillus subtilis,* and *Pichia pastoris* are commonly employed as expression hosts [10–12]. For example, BaPul was successfully expressed in *E. coli* in this study, and the yield of WT enzyme was about 26.6 mg/L. Compared with other hosts, *E. coli* possesses several distinct advantages, including its relatively simple manipulation, low cost, and rapid high-density cultivation, well-characterized genetics, large number of compatible tools, and ability to express foreign proteins abundantly; hence, this bacterium is the first choice for prokaryotic protein expression [13, 14]. However, two outstanding problems exist with pullulanase expression in *E. coli*, which include the following: (1) low level expression of soluble protein, and (2) poor extracellular secretion efficiency [15]. In recent years, researchers have proposed several strategies to improve soluble expression and secretion efficiency. Research has mainly focused on the regulation/optimization of fermentation process and optimization of the culture medium [16–18], but modification or engineering of a given protein has been rarely reported.

BaPul has been actively expressed in our laboratory in *E. coli* through a combination of vectors and hosts and considerable trial and error [19]. No pullulanase activity is detected in the medium, and the soluble form of total pullulanase is not very abundant, despite the optimization of fermentation parameters and codon usage. However, single-chain antibody fragments, which have low molecular weight and simple structure, can be expressed at higher soluble level and with higher secretion efficiency than BaPul using the same expression system and fermentation conditions (data have not been published). Therefore, we speculated that the high molecular weight and complicated structure of BaPul may hinder its soluble expression and secretion efficiency. Ramshini et al. in 2011 also indicated that proteins with high molecular weights and complicated structures have a propensity to form inclusion bodies, thus limiting their secretion [20].

Studies of other type I pullulanases from different species have shown that the N-terminal domain is not essential for industrial applications. Furthermore, an N-terminal domain truncation does not affect the debranching function against low molecular weight dextrins, while it retains secretion efficiency [21–23]. Therefore, we constructed different N-terminal truncated variants based on the BaPul 3D structure (PDB code 2WAN) in the present study. We subsequently investigated the soluble expression, secretion, and enzymatic properties of these variants.

## Methods

### Bacterial strains and plasmids

*Escherichia coli* BL21(DE3) was used as the recombinant pullulanase production strain. The plasmid pelB-pET28a(+) was used as an expression vector, which was constructed by replacing the fragment from *Bgl*II to *Xho*I of pET28a(+) with that of pET22b(+). The plasmid pMD18-T containing the full-length or truncated BaPul gene (Accession No. Ax203843.1) was used as the polymerase chain reaction (PCR) template.

### Genetic manipulation

The full-length and truncated pullulanase genes from *B*. *acidopullulyticus* were synthesized by Invitrogen (Shanghai, China) and ligated into the vector pMD18-T. These genes were amplified by PCR. Table 1 shows the primers used in this study. The PCR products were inserted into the *Nde*I/*Nco*I and *Xho*I sites of the expression plasmid. The resulting plasmids were verified by sequencing from the 5′ end of the inserted fragment.

**Table 1:** List of nucleotide sequences used in this study

Primer name	Nucleotide sequence (5′ → 3′)	Enzyme	Gene
WT[F]	GGGAATTC*CATATG*GATTCTACTTCGACTAAAGTTATTGTTC	*Nde*I	*Ba*Pul
M1[F]	GGGAATTC*CATATG*CCATCTGTTTCAAATGCCTATCTTG	*Nde*I	*Ba*Pul-ΔCMB41
M2[F]	GGGAATTC*CATATG*GATTCTACTTCGACTAAAGTTATTGTTC	*Nde*I	*Ba*Pul-ΔX45
M3[F]	GGGAATTC*CATATG*GATTCTACTTCGACTAAAGTTATTGTTC	*Nde*I	*Ba*Pul-ΔX25
M4[F]	GGGAATTC*CATATG*GTAACTGCCGTTCTTGTTGGAGATTTAC	*Nde*I	*Ba*Pul-ΔCMB41ΔX45
M5[F]	GGGAATTC*CATATG*CCATCTGTTTCAAATGCCTATCTTG	*Nde*I	*Ba*Pul-ΔCMB41ΔX25
M6[F]	GGGAATTC*CATATG*GATTCTACTTCGACTAAAGTTATTGTTC	*Nde*I	*Ba*Pul-ΔX25ΔX45
pelB-M1[F]	CATG*CCATGG*ATCCATCTGTTTCAAATGCCTATCTTG	*Nco*I	*Ba*Pul-ΔCMB41
pelB-M3[F]	CATG*CCATGG*ATTCTACTTCGACTAAAGTTATTGTTC	*Nco*I	*Ba*Pul-ΔX25
pelB-M5[F]	CATG*CCATGG*ATCCATCTGTTTCAAATGCCTATCTTG	*Nco*I	*Ba*Pul-ΔCMB41ΔX25
pelB-WT[F]	CATG*CCATGG*ATTCTACTTCGACTAAAGTTATTGTTC	*Nco*I	*Ba*Pul
[R]^a^	CCG*CTCGAG*TTGTTTGAGAATAAGCGTACTTATAG	*Xho*I	

Italicized fonts indicate the recognition sites of the corresponding restriction enzymes

*[F]* and *[R]* represent the forward and reverse primers, respectively

^a^[R] was used as the reverse primer in all cases

### Media and cultivation conditions

Luria–Bertani (LB) medium and modified Terrific broth (TB) medium supplemented with 50 μg/mL kanamycin or 100 μg/mL ampicillin was used for seed cultivation and shake flask cultures, respectively. LB medium contained 10 g/L tryptone, 5 g/L yeast extract, and 10 g/L NaCl. Modified TB medium contained 12 g/L tryptone, 24 g/L yeast extract, 2.31 g/L KH_2_PO_4_, 9.85 g/L K_2_HPO_4_, and 9.85 g/L glycerol (pH 7.0). Seed cultures were started by inoculating 10 mL of LB medium in a 100 mL shake flask with 10 μL of glycerol stock (stores frozen at −80 °C). The resulting culture was maintained at 37 °C for 10 h in a rotary shaker operating at 230 rpm. The seed culture (100 μL) was diluted in 10 mL of modified TB medium in a 100 mL shake flask at 20 °C and shaken at 230 rpm. After 5.5 h, 0.1 mM IPTG was added to induce target protein expression, and then incubation was continued for another 18–20 h.

### Cell fractionation

About 1 mL of culture broth was harvested via centrifugation at 4000×*g* and 4 °C for 30 min. The centrifuged supernatant was collected and defined as the extracellular fraction. The centrifuged cells were diluted in 1.0 mL of 10 mM PBS (pH 7.4) to a final OD_600_ of 4.0–5.0 and lysed by sonication (25 % amplitude, 2 s pulse with a 2 s interval between pulses, 6 min in total) on ice. The intracellular fraction was isolated by centrifugation of the ultrasonic product at 12,000×*g* and 4 °C for 10 min. The resulting cell debris was obtained as the insoluble intracellular fraction.

The centrifuged cells were resuspended in 1 mL of 30 mM Tris–HCl buffer (pH 8.0) containing 20 % (wt/vol) sucrose and 1 mM EDTA at 4 °C overnight (8–10 h); these cells were subsequently centrifuged at 4000×*g* for 30 min. The obtained supernatant was the periplasmic fraction I. The resulting pellet was resuspended in 1 mL of 5 mM MgSO_4_ buffer on ice for 10 min and centrifuged at 4000×*g* for 20 min. The obtained supernatant was the periplasmic fraction II. The sum of the periplasmic fraction I and II was defined as the periplasmic fraction. The obtained precipitate was resuspended in 10 mM PBS (pH 7.4) and disrupted by sonication. After centrifugation at 12,000×*g* and 4 °C for 10 min, the supernatant was collected as the cytoplasmic fraction.

### Enzyme activity assay

Pullulanase activity was measured by incubating the enzyme at 60 °C for 10 min with 1 % pullulan. The activity was determined by assaying the release of reducing sugars via the 3,5-dinitrosalicylic acid method [19]. One unit of enzyme activity is defined as the amount of enzyme required to release 1.0 μmol of reducing sugars (with glucose as the standard) per minute under the specified assay conditions. The total pullulanase activity is the sum of enzyme activity in the extracellular and intracellular fractions.

### SDS-PAGE analysis

SDS-PAGE was performed using a 5 % stacking gel and 9 % (or 12, or 15 %) separating gel. The protein bands were visualized by staining with Coomassie brilliant blue R-250.

### Protein purification and quantification

The enzymes were purified with an AKTA purifier system (GE Healthcare, Sweden). The cell pellets of the overexpression product were resuspended in binding buffer (20 mM sodium phosphate, pH 7.6). The solution was supplemented with 1 mM phenylmethylsulfonyl fluoride and disrupted by sonication. The cell debris was removed by centrifugation at 4 °C at 15,000×*g* for 10 min. The purified enzyme solution was filtered (0.22 μm filter) before loading onto a 1 mL of HiTrap Capto Q anionic chromatography column and then washed with 10 mL of binding buffer. The enzyme was eluted with an elution buffer (20 mM sodium phosphate, 1 M NaCl, pH 6.0). The fraction containing the target enzyme was loaded onto a HisTrap HP affinity column for further purification as previously described [33]. Enzyme purity was monitored by SDS-PAGE analysis. Enzyme concentrations were measured using the Bradford method (Coomassie Brilliant Blue G-250 method) with bovine serum albumin as the standard [34].

### Bacterial biomass

Bacterial biomass was monitored at the end of the cultivation period by measuring the optical density of culture broth at 600 nm using a spectrophotometer (BioPhotometer Plus; Eppendorf Co., Hamburg, Germany). Samples were diluted with 0.9 % (w/v) NaCl as necessary to maintain the readings within the 0.2–0.8 absorbance unit range.

### Optimum pH and temperature

Optimum pH for pullulanase activity was determined by assaying the enzyme activity in buffers with different pH values that ranged from 3.0–7.0 at 60 °C in glycine–HCl (pH 3.0, 100 mM), citric acid–sodium citrate (pH 3.5 and 5.5, 100 mM), sodium acetate–acetic acid (pH 4.0–5.0, 100 mM), or Na_2_HPO_4_–NaH_2_PO_4_ (pH 6.0–7.0, 100 mM). The optimum temperature for pullulanase was determined by assaying the enzyme activity in 100 mM sodium acetate buffer (pH 5.0) at different temperatures (40–80 °C).

### Application

Starch slurry (15 L) with 30 % solid fraction (dry weight) was prepared with a pH value of 5.5–6.2, with α-amylase (10–15 U/g of solid substrate) and calcium (1 mM). The liquefaction process was carried out at 72 °C for 15 min, followed by 95 °C for 60 min. Prior to the saccharification, the pH was reduced to 4.2–4.5, hence maintaining a high temperature (above 95 °C) for 30 min to inactivate the liquefying α-amylase. The temperature was then lowered to 60 °C, glucoamylase (120 U/g of solid substrate) and pullulanase (0.5 U/g solid substrate) were added, and the saccharification proceeded for about 24–72 h. The saccharification was completed when no dextrin could be detected with anhydrous alcohol. The liquid was boiled for 10 min in water to inactivate the enzymes [11]. The dextrose equivalent was defined as the reducing sugars (with glucose as the standard) versus the solid substrate.

## Results

### Design of N-terminal domain truncated variants

**Fig. 1:**
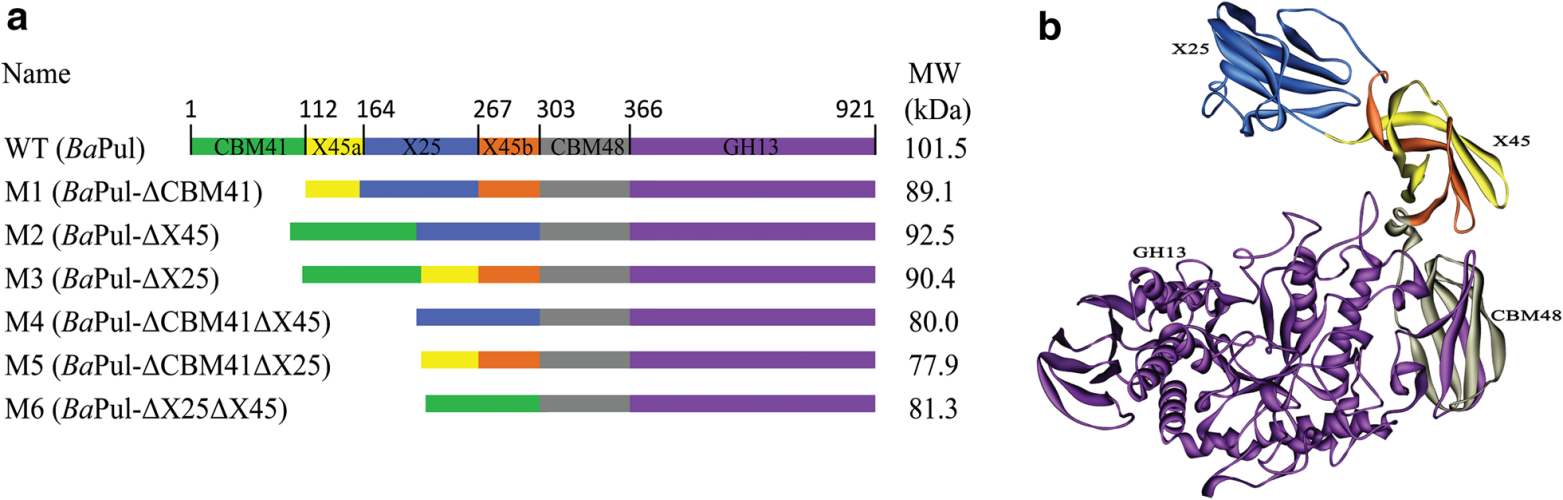
Schematic representation of the wild-type (WT) and variants. **a** The primary structure of the WT and variants. Numbers represent the first residues of each domain. Different *colors* represent different domains. The names of the variants are indicated on the *left*. The predicted molecular sizes are indicated on the *right*. **b** 3D structure of BaPul (PDB code 2WAN). The CBM41 domain is disordered in-crystal and cannot be modeled. The figure was produced using Discovery Studio 2.5 (Accelrys, USA)

BaPul is a mature protein of 921 amino acids and has a complex modular architecture. Its structure consists of CBM41-X45a-X25-X45b-CBM48-GH13, where CBM is a carbohydrate-binding domain, X is a module of unknown function, and GH13 indicates a domain of *CA*rbohydrate-active en*ZY*mes (CAZY, see http://www.cazy.org). GH13 possesses a classical (β/α)_8_ barrel 3D structure and consists of classical A, B, and C domains of generic α-amylases [24]. The crystal structure shows that the CBM48 domain is closely connected with the GH13 catalytic module. This characteristic is similar to isoamylase. The CBM48 domain and GH13 catalytic module may retain full catalytic function of the debranching enzyme. Three N-terminal domains, namely CBM41, X45, and X25, are not in close contact with GH13 catalytic module, which may be associated with the binding of high-molecular-weight polysaccharides. We designed six N-terminal truncated variants according to the 3D structure of BaPul (Fig. 1).

### Detection of soluble expression of the WT and variants

**Fig. 2:**
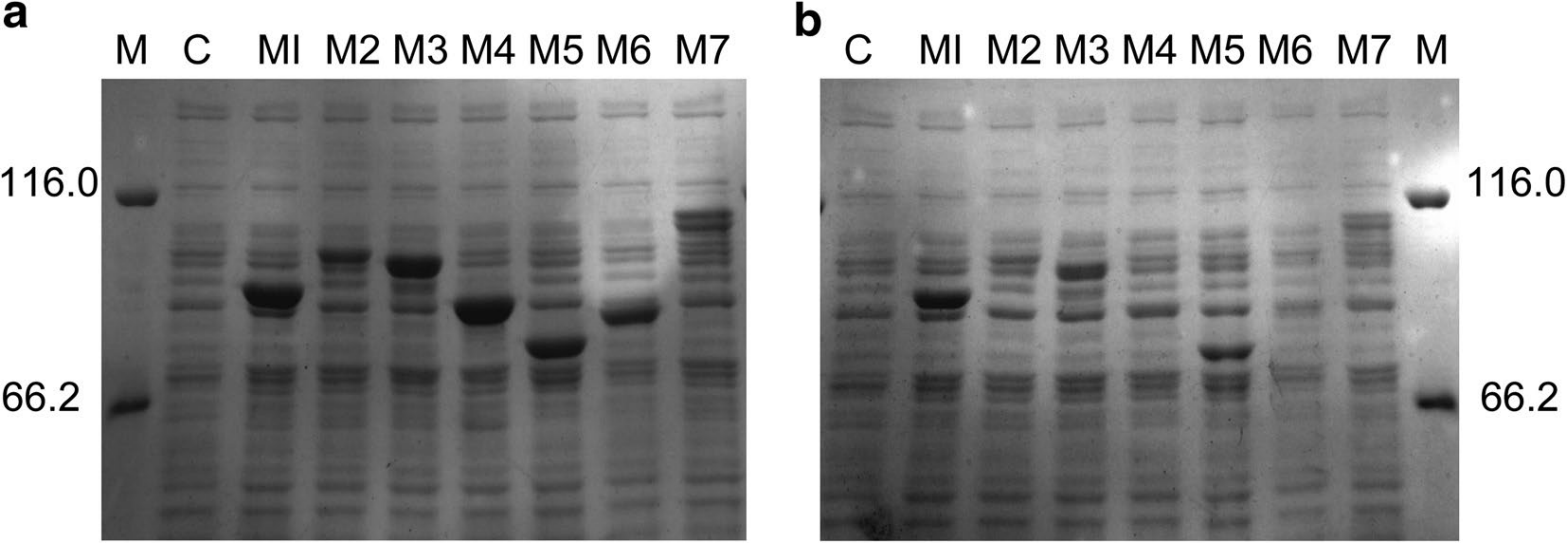
SDS-PAGE analysis of the WT and variants. Lanes: M, protein molecular mass markers; *C* control [vector pET28a(+) without foreign gene]; *M1*; *M2*; *M3*; *M4*; *M5*; *M6*; *WT*. The values are listed as molecular sizes in kilodaltons. **a** Total cellular protein. **b** Soluble fraction of total cellular protein

The genes encoding the WT and variants were subcloned into the vector pET28a(+) and expressed in *E. coli* BL21(DE3). No pullulanase activity was detected in the extracellular fraction. All of the target proteins were located in the cytoplasm because of the lack of a signal peptide. The pullulanase activities of M1 and M5 (both lack the CBM41 domain), which were 2.9- and 2.4-fold that of the WT, respectively, were higher than the other recombinant strains. The pullulanase activity of M3 was similar to WT activity. No activity was detected for M2 (ΔX45), M4 (ΔCBM41ΔX45), or M6 (ΔX25ΔX45) in any fraction (Table 2). These three recombinant strains often lack the X45 domain. Sodium dodecyl sulfate–polyacrylamide gel electrophoresis (SDS-PAGE) results showed that all of the target proteins were expressed in *E. coli*. The soluble expression levels of M1 and M5 were higher than those of the WT and M3. Almost all of the target proteins for M2, M4, and M6 variants were expressed in inclusion bodies (Fig. 2). Subsequent experiments did not involve these three variants.

**Table 2:** Pullulanase activity of the WT and variants

Variants	IA (U/mL)	TA (U/mL)
M1 (ΔCBM41)	84.6 ± 2.6	84.6 ± 2.6
M2 (ΔX45)	–	–
M3 (ΔX25)	35.7 ± 1.1	35.7 ± 1.1
M4 (Δ45ΔX25)	–	–
M5 (ΔCBM41ΔX25)	71.0 ± 2.4	71.0 ± 2.4
M6 (ΔCBM41ΔX45)	–	–
Wild-type	29.1 ± 0.5	29.1 ± 0.5

*IA* intracellular activity; *TA* total activity

– Below the detection line

These results indicate that variants lacking the CBM41 domain can demonstrate improved pullulanase activity. Removal of the X25 domain exhibited almost no effect on pullulanase activity, whereas removal of the X45 domain resulted in the formation of the inclusion bodies. The X45 domain may contain information within its amino acid sequence that facilitates catalytic module folding to obtain the correct conformation [21].

### Improved activity was caused by improved soluble expression

**Fig. 3:**
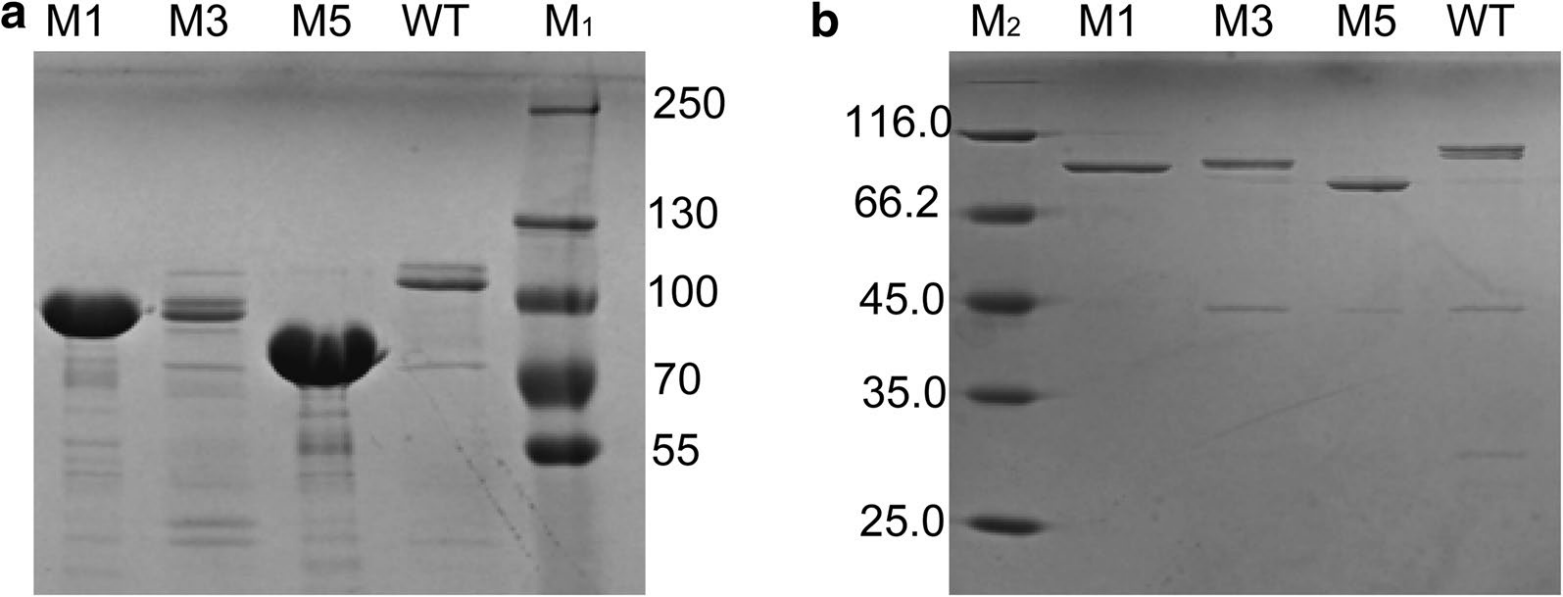
SDS-PAGE analysis of the WT and variants. Lanes: *M*_*1*_ and *M*_*2*_, protein molecular mass markers; *M1*; *M3*; *M5*; *WT*. The values between the panels are listed in molecular sizes in kilodaltons. **a** Anionic chromatography purification on HiTrap Capto Q column (GE Healthcare, Sweden). **b** His-tag purification on complete His-Tag Purification Column (GE Healthcare, Sweden)

To investigate the reasons for activity improvement, we purified the soluble target proteins, and then used the purified proteins to measure the specific activity and protein concentration. Anionic chromatography was conducted on a HiTrap Capto Q column for primary purification of 10 mL of culture broth. Apparently, the soluble expression levels of M1 and M5 were higher than those of M3 and the WT (Fig. 3a). Further purification using a Ni^+^ column revealed a single target protein band on an SDS-PAGE gel, which could be used for specific activity measurement (Fig. 3b). Specific activity results indicated a slight difference between the WT and variants (Table 3). Therefore, the ratio of total enzyme activity appears to reflect the ratio of the soluble expression level. Protein quantification results also indicated the evident differences in the soluble expression levels of M1 (72.5 mg/L) and M5 (55.9 mg/L), which were 2.7- and 2.1-fold that of the WT (26.6 mg/L), respectively (Table 3). The fold of activity improvement for M1 and M5 was similar to the fold of expression level improvement. These results show that the improvement in pullulanase activity was the resulted from enhanced soluble expression.

**Table 3:** Specific activity and protein concentration of the WT and variants

Variants	Protein concentration^a^ (μg/mL)	Specific activity (U/mg)
M1 (ΔCBM41)	72.5 ± 2.2	1167.3 ± 35.0
M3 (ΔX25)	31.7 ± 0.7	1124.4 ± 24.7
M5 (ΔCBM41ΔX25)	55.9 ± 1.8	1271.1 ± 41.9
Wild-type	26.6 ± 0.5	1096.5 ± 21.9

^a^Soluble fraction

### Proteins lacking the CBM41 domain were located in extracellular fraction with PelB

Although the soluble expression levels of M1 and M5 were improved compared with that of the WT, a larger number of target proteins were expressed in inclusion bodies. Inclusion bodies are widely considered the nonspecific aggregation of incompletely folded or partially denatured polypeptides when the recombinant protein is highly expressed [25–27]. Several studies have shown that secretion of recombinant protein into the periplasmic space or extracellular medium is helpful for the correct folding of the protein, which increases its soluble expression [13]. The genes encoding the WT enzyme and variants were subcloned into the vector pET28a(+)-pelB and expressed in *E. coli* BL21(DE3). No pullulanase activities were detected for PelB-WT and PelB-M3 in the extracellular medium, whereas those for PelB-M1 and PelB-M5 were 12.8 and 62.7 % of the total activity, respectively. The PelB signal peptide exhibited no active effect on soluble expression level, but facilitated secretion. The pullulanase activities in the periplasmic space for PelB-M1 and PelB-M5 were 43.8 and 30.7 %, respectively (Table 4). Almost all of the soluble target proteins of PelB-WT and PelB-M3 were located in the cytoplasm.

**Table 4:** Pullulanase activity of the WT and variants

Variants	EA (U/mL)	PA (U/mL)	CA (U/mL)	TA (U/mL)	(EA/TA): (PA/TA): (CA/TA)
pelB-M1 (ΔCBM41)	10.7 ± 0.4	36.7 ± 0.8	36.3 ± 0.9	83.7 ± 2.1	12.8: 43.8: 43.4
pelB-M3 (ΔX25)	–	1.3 ± 0.4	29.1 ± 0.7	30.4 ± 1.1	–: 4.3: 95.7
pelB-M5 (ΔCBM41ΔX25)	46.9 ± 1.2	23.0 ± 0.6	4.9 ± 0.5	74.8 ± 2.3	62.7: 30.7: 6.6
pelB-Wild-type	–	1.2 ± 0.4	30.7 ± 0.6	31.9 ± 1.0	–: 3.8: 96.2

*EA* extracellular activity, *PA* periplasmic space activity, *CA* cytoplasmic activity, *TA* total activity

– Below the detection line

### Cell lysis led to the improvement of extracellular activity

**Fig. 4:**
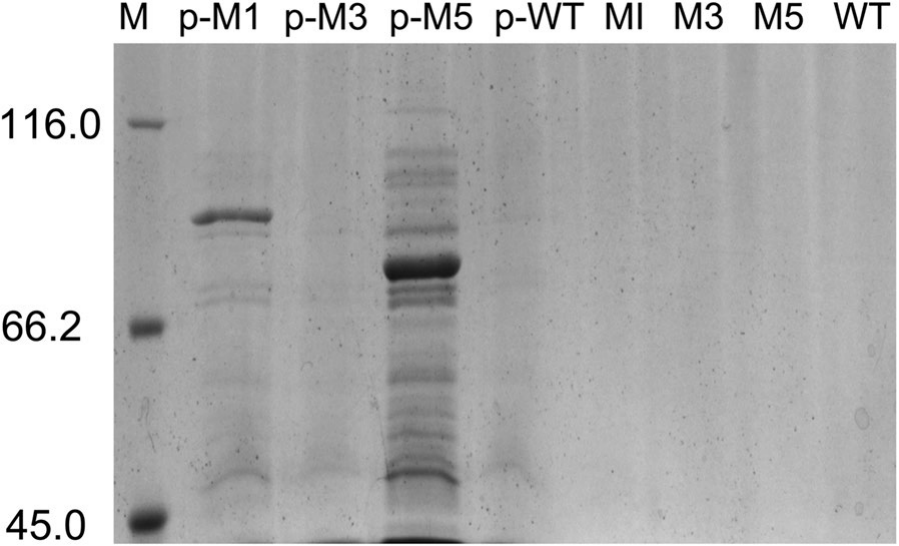
Extracellular medium SDS-PAGE analysis of the WT and variants. Lanes: *M*, protein molecular mass markers; *p-M1*, *pelB-M1*; *p-M3*, *pelB-M3*; *p-M5*, *pelB-M5*; *p-WT*, *pelB-WT*; *M1*; *M3*; *M5*; *WT*. The values are listed as molecular sizes in kilodaltons

The cells lysis of pelB-M1 and pelB-M5 occurred at the end of the parallel fermentation, which was specifically apparent for pelB-M5. The experimental phenomena were viscous culture broth, cell pellets, a sharply declining OD_600_, and difficulty in centrifugation. The OD_600_ of pelB-M1, pelB-M3, pelB-M5, and pelB-WT were 14.0, 17.2, 10.2, and 16.9, respectively (Table 5). The results of SDS-PAGE gels also confirmed that the cell lysis of PelB-M1 and PelB-M5 led to intracellular protein release (Fig. 4). However, the differences in the OD_600_ between M1, M3, M5, and the WT in the same batch were not evident at the end of the fermentation (Table 5). Moreover, no protein was found in the extracellular medium. Figure 4 and Table 5 confirm that cell lysis of PelB-M1 and PelB-M5-containing strains leads to improved extracellular activity. Furthermore, the results did not exclude nonspecific leakage, which may also improve the extracellular activity.

**Table 5:** OD_600_ of the WT and variants at the end of the fermentation

Variants	OD_600_	Strains	OD_600_
pelB-M1 (ΔCBM41)	14.0 ± 0.5	M1 (ΔCBM41)	18.7 ± 0.4
pelB-M3 (ΔX25)	17.2 ± 0.2	M3 (ΔX25)	18.6 ± 0.6
pelB-M5 (ΔCBM41ΔX25)	10.2 ± 0.4	M5 (ΔCBM41ΔX25)	19.2 ± 0.3
pelB-Wild-type	16.9 ± 0.1	Wild-type	18.1 ± 0.5

### Effects of pH and temperature on the activities of the WT and variants

**Fig. 5:**
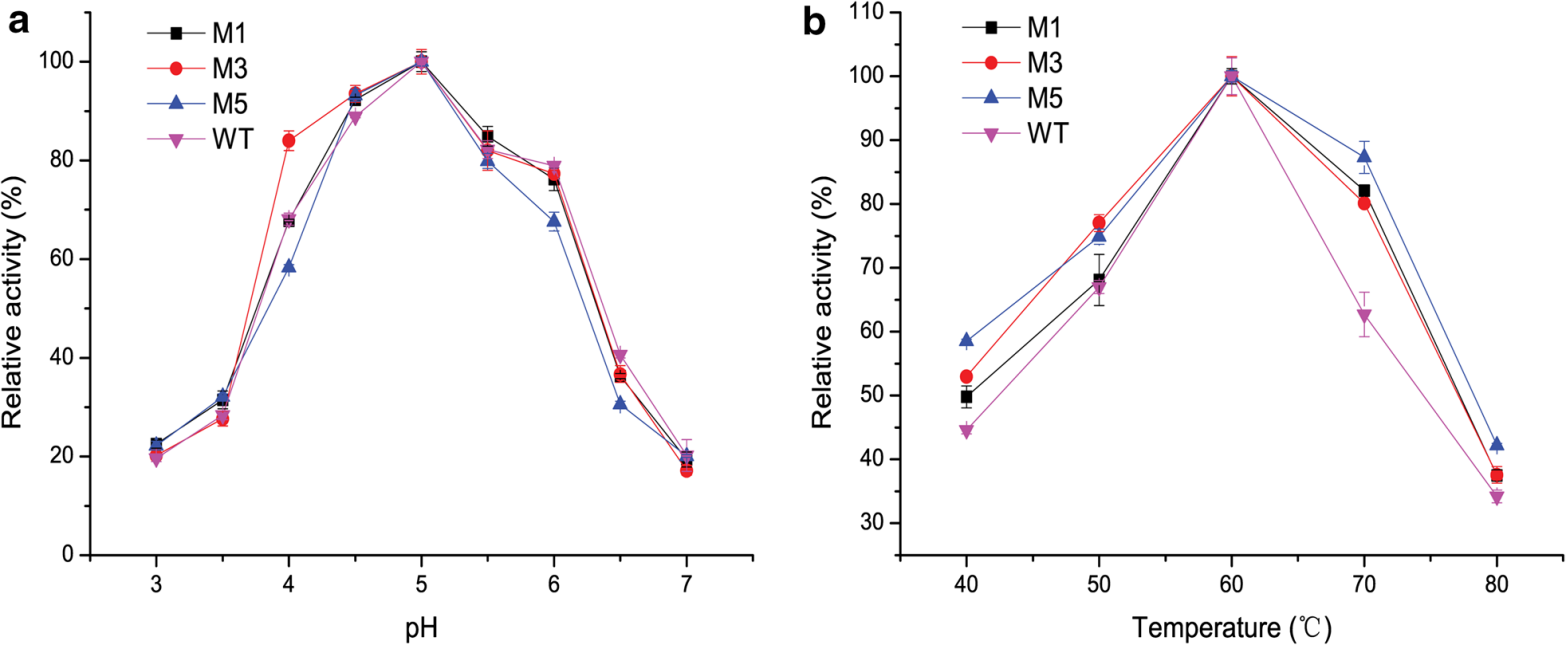
Effect of pH and temperature on the activities of the WT and variants. **a** pH. Assays were carried out at 60 °C for 10 min in buffers with pH values within the range of 3.0–7.0. **b** Temperature. Assays were conducted in 100 mM sodium acetate–acetic acid buffer (pH 5.0) within the temperature range of 40–80 °C for 10 min

The optimal pH and temperature for M1, M3, and M5 were 5.0 and 60 °C respectively, which are similar to the optimal values for the WT (Fig. 5). The relative activities of M1, M3, M5, and WT (without pelB leader) at 70 °C were 82, 80, 87, and 63 %, respectively. These results show that the variants had better heat resistance than the WT at 70 °C. Truncation exhibited minimal effect on the adaptation to pH; the pH profiles of the WT and variants were nearly the same.

### Application

After the liquefaction step, the long-chained starch was degraded into smaller branched and linear maltodextrins, with a dextrose equivalent of 12–15 %. At the end of the saccharification step, the dextrose equivalents of M1, M3, and M5 variants were 94.7, 94.5, and 93.1 %, respectively, which were essentially identical to those of the WT (93.6 %). Therefore, the deletion of the CBM41 domain and/or X25 domain did not affect the practical application of the enzyme to the starch saccharification process.

## Discussion

### Different variants have different soluble expression levels

We found that the soluble expression level of different variants varied remarkably when expressing different pullulanase variants using the same *E. coli* system. This phenomenon was not dependent on the experimental conditions. All of the pullulanase variants were subjected to the same most suitable conditions of 20 °C and 0.1 mM isopropyl-β-D-thiogalactopyranoside (IPTG). Most of the target proteins were expressed in inclusion bodies, but the protein amount in the soluble fractions from M1 and M5 was increased. The soluble expression level can likely be further improved via optimization of the fermentation conditions and medium in a future study.

**Fig. 6:**
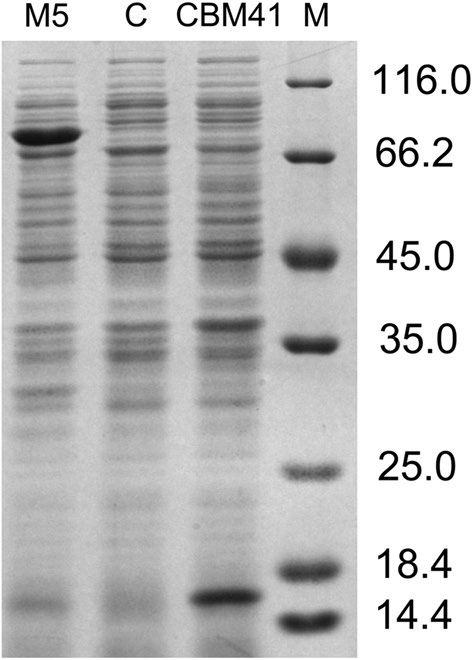
SDS-PAGE analysis of the soluble fraction. Lanes: *M*, protein molecular mass markers; *M5*; *C* control (empty vector); *CBM41*. The values are listed as molecular sizes in kilodaltons

Comparing M1, M3, M5, and the WT, we found that the soluble expression level of M3 and the WT (retaining the CBM41 domain) was lower than that of M1 and M5 (lacking CBM41 domain). The CBM41 domain appears to be an adverse factor that affects soluble protein expression. This domain is expressed separately, and M5 and the empty vector are used as controls (Fig. 6). SDS-PAGE results showed that the soluble expression level of CBM41 was comparable with M5. Thus, we speculated that the amorphous structure of CBM41 domain makes the entire structure looser with a higher molecular weight, which make M3 and the WT more likely to be expressed in inclusion bodies compared with M1 and M5.

### Different variants have different secretion efficiencies

The protein carrying the PelB signal peptide is generally secreted into the periplasmic space by the PelB transport protein, and only a small amount of the protein is released into the culture medium via nonspecific leakage [28]. However, the signal peptide is necessary but insufficient for the transmembrane transport of cytoplasmic protein to the extracellular medium [29]. For example, no more than 5 % of the target protein of PelB-WT and PelB-M3 was observed in the periplasmic space. PelB cannot effectively guide WT and M3 transport from the inner membrane into the periplasmic space, and more than 95 % target proteins were retained in the cytoplasm. The main reason for retention may be related to the molecular weight and structure of the protein. The smaller molecular weight of the protein resulted in larger degree of transmembrane transport. The predicted molecular sizes of MI and M3 were almost the same, but M1 had a higher secretion efficiency than M3. We speculated that proteins with non-compact structures may also have lower secretion efficiencies.

PelB was active and stimulated cell lysis for PelB-M1 and PelB-M5. The overexpression of M1 and M5 proteins, which were transported into the periplasmic space under PelB guidance, may quickly jam this narrow space, thereby hindering the exchange of materials inside and outside the cell. Consequently, cell apoptosis or spontaneous lysis may occur, hence releasing the intracellular proteins.

### Protein engineering is an effective method to improve the soluble expression and secretion efficiency of given proteins

We compared this study with those found in PubMed database. We found that the soluble expression level and extracellular secretion efficiency of different proteins vary significantly, even when the same expression system and fermentation conditions are employed [13, 21]. The amino acid sequence, 3D structure, and quaternary structure of the foreign protein are important factors that affect the expression level and secretion efficiency [29, 30]. Some progress can be observed in increasing soluble expression and secretion efficiency by using protein engineering [31, 32]. We proposed to conduct a research to investigate the potential correlation between expression levels and extracellular secretion efficiency of recombinant proteins and their amino acid sequence or structures on the basis of heterologous expression in *E. coli*.

In the present study, we improved the soluble expression and extracellular secretion efficiency of pullulanase via protein engineering. The soluble expression level is at least partially related to inclusion body formation. However, the mechanism for the formation of inclusion bodies is not clearly elucidated. The reason for the increase in soluble expression is only an inference based on our experimental results. Notably, our analysis results differ from similar studies. For example, a patent by Genecor in 2008 and study by Duan et al. in 2015 proposed that the increased extracellular secretion efficiency of truncated pullulanase variants may be related to their low molecular weights [21, 22]. We partially agree with the speculation that low-molecular-weight proteins may be secreted because of non-specific leakage. However, we propose that the release of intracellular proteins of pelB-M1 and pelB-M5 via cell lysis is the main reason for improved extracellular activity.

## Conclusions

To improve the soluble expression level and secretion efficiency of BaPul in *E. coli*, we created N-terminal domain truncations. Deletion of the CBM41 domain could effectively improve the enzymatic activity yield, and deletion of X25 had minimal effect on the activity yield. The loss of the X45 domain resulted in reduced catalytic activity. Moreover, and the target protein mainly existed in inclusion bodies. The specific activities of the truncated mutant and WT enzymes were similar, and the main reason for the improved activities was be the enhanced soluble expression of pullulanase. Under the guidance of PelB signal peptide, the variants lacking the CBM41 domain can be secreted to the periplasm, followed by cell lysis and leakage of the enzyme to the extracellular medium. Furthermore, the deletion of the CBM41 and/or X25 domain did not affect the actual application in starch saccharification. Therefore, several of these variants have potential applications in industrial processes, as they can be more readily purified than the WT enzyme. This work also provides the foundation for further advances in recombinant pullulanase technology.
